# Potential Therapeutic Properties of the Leaf of *Cydonia Oblonga* Mill. Based on Mineral and Organic Profiles

**DOI:** 10.3390/plants11192638

**Published:** 2022-10-07

**Authors:** Diana Melo Ferreira, Natália M. de Oliveira, Lara Lopes, Jorge Machado, Maria Beatriz Oliveira

**Affiliations:** 1 LAQV/REQUIMTE—Department of Chemical Sciences, Faculty of Pharmacy, University of Porto, 4050-313 Porto, Portugal; 2 Laboratory of Applied Physiology, Institute of Biomedical Sciences Abel Salazar, University of Porto, 4050-313 Porto, Portugal; 3 Centre of Biosciences in Integrative Health, 4250-105 Porto, Portugal

**Keywords:** *Cydonia oblonga* Mill. leaves, inorganic composition, phenolic compounds, vitamin E, fatty acids, nutraceutical potential

## Abstract

Leaf extract of *Cydonia Oblonga* Mill. is interesting for further exploration of the potential of its substrates for therapeutic supplements. Quantitative and qualitative analyses were conducted on samples of green (October), yellow (November), and brown (December) quince leaves collected in the region of Pinhel, Portugal. Mineral analysis determined the measurements of the levels of several macro- and micro-elements. Organic analysis assessed the moisture content, total phenolic content (TPC), vitamin E, and fatty acid (FA) profiles. Mineral analysis was based on ICP-MS techniques, while the profiles of vitamin E and FA relied on HPLC-DAD-FLD and GC-FID techniques, respectively. Moisture content was determined through infrared hygrometry and TPC was determined by spectrophotometric methods. Regarding the mineral content, calcium, magnesium, and iron were the most abundant minerals. Concerning organic analysis, all leaf samples showed similar moisture content, while the TPC of gallic acid equivalents (GAE) and total vitamin E content, the most predominant of which was the α-tocopherol isomer, showed significant variations between green-brown and yellow leaves. FA composition in all leaf samples exhibited higher contents of SFA and PUFA than MUFA, with a predominance of palmitic and linolenic acids. Organic and inorganic analysis of quince leaves allow for the prediction of adequate physiological properties, mainly cardiovascular, pulmonary, and immunological defenses, which with our preliminary in silico studies suggest an excellent supplement to complementary therapy, including drastic pandemic situations.

## 1. Introduction

The quince of Persia is a plant also known as *Cydonia vulgaris* Persoon or *Cydonia oblonga* Miller, and is a monotypic genus comprising the family Rosaceae, subfamily Maloideae, and genus Cydonia (USDA, 2009) [1]. Currently coined with different names—the Arabic name “Sefarjal”, the Azari name “Heyva”, the Urdu name “Bahee Dana”, the Farsi name “Beh”, the Hindi name “Bihi”, the Italian name “Cotogno”, the German name “Quitte”, the Spanish name “Membrillo”, the Portuguese name “Marmeleiro” and the French name “Cognassier or Pommier de Cydon”—the etymology derives from the Latin cotoneum mālum (quince fruit), probably a variant of cydonium malum from the Greek kydōnion malon, which is traditionally referred to as the “apple of Kydōnia” (modern-day Greek city of Chania), a prominent ancient seaport city on the Northwest coast of Crete [2,3]. Regarding its taxonomy and plant characteristics, the quince plant grows as a shrub or small tree (4–6 m high) and is rounded by a canopy up to 3 m in diameter. The root presents a superficial and fasciculate system with a tortuous trunk. The leaves are green-intense and bright, with a shape from oval to oblong (5–10 cm in length across, 5 cm width). The large and single flowers (4–5 cm) vary from white to pink shades. Each flower with five petals, five styles, 20 or more stamens, and a lower ovary with many ovules reaches full blossom during the respective spring season of each hemisphere [4,5]. The fruits ripen during autumn, depending on the climatic conditions of the habitats on which they are farmed. They are covered with grey-whitish hairs and may reach a weight of about 300–500 g. The quince fruit can be left on the tree to ripen further, which softens the fruit to the point where it can be eaten raw in warmer climates but should be picked before the first frosts, at a time when the entire surface of the fruit is pale yellow and is easy to tear it off by twisting the stalk. Three common varieties of *Cydonia oblonga* Miller exist in Europe: *Cydonia vulgaris* Maliformis, *Cydonia vulgaris* Pyriformis, and *Cydonia vulgaris* Lusitanica. *Cydonia vulgaris* Lusitanica is the Lusitanian or Portugal quince. This variety has broader leaves and larger fruits than the former two, and the fruit has a pear shape similar to *Cydonia vulgaris* Pyriformis, and stands out by its robust growth and improved adjustment for stocks to graft upon. It is less valuable as a bearer compared to the other two varieties and the fruit’s skin is deep yellow instead of orange. Regardless, this variety is considered of high value for marmalade, as its pulp turns to fine purple and becomes milder and less austere due to the formation of anthocyanins when stewed or baked [6]. The pulp is yellowish, acidic, astringent, and consumed processed as jams, jellies, marmalade, and cakes. This cultivar was taken to England by John Tradescant in 1611, when it was described as the best quince for baking [7]. With respect to the the origin and geographical distribution, this plant is native to the northern forests of Asia Minor and the Caucasus Mountains between Persia and Turkmenistan, though quince can still be found in the wild form in Dagestan, Azerbaijan, Turkmenia, and Iran [8]. There are records of quinces being cultivated 5,000 years ago in Mesopotamia (now Iraq), and from 100 BC onwards, they were popular in Palestine long before apples [9]. From the Central Asia region, the plant crossed old trade routes to the east and west, and it was first noticed within southwest Europe on the ancient island of Crete and most probably reached the Mediterranean during the classical (Greco–Roman) times. Due to its medicinal properties, quince has been known since ancient times in mythology and folk tradition as a symbol of love, happiness, fertility, wisdom, beauty, durability, and eternity. The Plutarch of Chaeronea in Boeotia (45–120 AD), a Platonist author, confirms this when he reports the tradition of sharing a quince between a couple of newlyweds as a harbinger for the couple’s fertility. Additionally, Romans cultivated quinces for their medicinal qualities. A terracotta life-sized quince found in in Apulia in southern Italy was traced back to 300–250 BC. *De Agricultura* of Marcus Porcius Cato the Elder (234–149 BC), a farming manual on agriculture of 202 BC, recommended growing three types of quinces that ripened well: Strutea, Cotonea, and Mustea. Pliny the Elder (23–79 AD), a Roman naturalist, praised their medicinal virtues and discriminated the Mulvan variety, which was the only cultivated quince at the time that could be eaten raw. Furthermore, Columella (4–10 AD), a Roman agriculture writer says: *“Quinces not only yield pleasure, but health.”* [10]. Both Greeks and Romans preserved quinces in honey, giving rise to the name *melimelum* from the Greek for honey apple, which evolved into the Spanish marmello and presently, membrillo and Portuguese marmelo. They are rich in pectin, which allows them to set as jam or jelly when cooked. This quality was first discovered by the Romans, who cooked the fruit prior to preserving it. Quinces were the only fruits that needed cooking first, and for a long time it was assumed that they were the only fruits that would set in this way. Portuguese quince jam or marmelada (from the Portuguese marmelo) reached Britain in the 16th century and became a popular way to preserve fruit. Apart from its historical importance, it still carries an economic value with cultivation worldwide, where Turkey holds 25% of the world production followed by China, Iran, Argentina, and Morocco, each producing less than 10%. The United States is a very minor producer of quince fruit, mainly in California’s San Joaquin Valley [11]. In Portugal, *Cydonia vulgaris* Lusitanica is widely spread in the northeast, central east, and southern regions such as Trás-os-Montes, Beira Alta, and Ribatejo, respectively. It is also possible to find it in the Minho and Beira Litoral regions. Several parts of the plant have functionality and have been constitutionally used for different purposes: wood of quince is used for furniture making, the fruits and its juice are rich in thiamine, riboflavin, nicotinic acid, vitamin B6, inositol, pantothenic acid, folic acid, and biotin [12]. Quince fruit may be consumed raw in some countries where population is used for astringent flavors [13], such as in South America, and cooked quince is still used as a complement to apple pies and brandy, for jam and marmalade making, and even as tea [14]. Aromatic and functional properties of quince have also been used to fortify products such as beers and yogurts. Moreover, the seed mucilage of this quince fruit, a hydrocolloid, has been useful as a bulking and thickening agent in food [15]. The medicinal properties of this plant reported during ancient times caught the eye of modern scientific researchers that have looked up to isolation of plant active phytochemicals [16,17,18,19,20,21,22,23], using their curative potential in response to health challenges [24,25,26,27,28,29]. In the past few years, the chemical constituents of quince fruit and its derivatives have been the subject of study [13,30,31,32,33,34,35,36]. In the leaf of the Portuguese quince, several phytochemicals have been isolated, from which phenolic compounds such as organic acids (oxalic, citric, malic, quinic, shikimic, and fumaric acids); caffeoylquinic acids (3,4,5-O-caffeoylquinic acids, 3,5-O-dicaffeoylquinic acid) [33]; and flavonoids (quercetin-3-O-galactoside, quercetin-3-O-rutinoside, kaempferol-3-O-glycoside, kaempferol-3-O-glucoside, and kaempferol-3-O-rutinoside) [31,32] were identified. Similarly, quercetin-3-O-galactoside, quercetin-3-O-rutinoside, kaempferol-3-O-glycoside, kaempferol-3-O-rutinoside and kaempferol-3-O-glucoside, and 4-O-caffeoylquinic acid were identified in the methanolic extract of Tunisian Quince leaves [37]. Rutin (36%) was the most abundant flavonoid found in its leaves [38]. On the other hand, chlorogenic acid was identified as the major phenolic compound in Quince leaf methanolic extract [39]. Similar to phenolic compounds, organic acids are important secondary metabolites of plants with antioxidant properties. Quince leaves from central and northern Portugal contained quinic acid (72.2%), oxalic acid (6.1%), malic acid (7.6%), and citric acid (13.6%) with small amounts of fumaric and L-shikimic acids. It was also observed that greener leaves harvested earlier in June and August had higher acid contents than those collected in October [33]. Leaves gathered during the flowering and fruiting seasons had a composition of 47 and 40 different essential oils each, composing 95.7% and 64.5% of their respective total oils. Aldehydes were 12.8% of the total oils in the leaves of the flowering period, followed by fatty acids (7.2%), monoterpenes (5.7%), and norisoprenoids such as (*E*)-β-Ionone (5.1%) and (*E,E*)-α-Farnesene (4.6%). However, the leaves of fruiting quince contained hydrocarbons of sesquiterpene (8.6%), benzaldehyde (4.9%), and (*Z*)-β-Farnesene (4.8%) as the main constituents of essential oils [40]. Facing this difference in composition, the present study aimed to determine the chemical composition of Portuguese quince leaves using a more recent collection from the largest region with certified biological quince cultivars in Europe (an area of about 800 acres) managed by the Biocôa Association in the Pinhel region. To understand the functional and therapeutic properties of this part of the plant and its potential applications in different industries, the assessment of the chemical composition of the leaf of *Cydonia oblonga*, a variety *Cydonia vulgaris* Lusitanica, analysis of the inorganic (mineral composition) and organic profiles (moisture content, total phenolic content, vitamin E, and fatty acids profiles) were accomplished. The possible pharmaceutical properties associated with risk reduction in aging processes and the development of chronic diseases such as cardiovascular, pulmonary, and cancer were also considered. Sample collection happened in two different periods of maturation: A first sample of green leaves collected in October, just before the fruit harvest, another of yellow leaves collected after the fruit harvest in November, and a third collection of brown leaves in December. Additionally, in silico studies with clinical cases (preliminary personal data) showed distinct but complementary therapeutic actions between green, yellow, as well as brown quince leaves.

## 2. Materials and Methods

### 2.1. Chemical Reagents

Absolute ethanol was obtained from Fisher Chemical (Loughborough, England). methanol, gallic acid, Folin–Ciocalteu reagent, sodium carbonate (Na_2_CO_3_), and 1,4-dioxane were from Sigma (St. Louis, MO, USA). HNO_3_ (Merck^®^) and H_2_O_2_ (Merck^®^). Potassium hydroxide (KOH), anhydrous sodium sulfate (Na_2_SO_4_), and n-hexane (HPLC grade) were obtained from Merck (Darmstadt, Germany). Tocol (2-methyl-2-(4,8,12-trimethyl-tridecyl) chroman-6-ol) was obtained from Matreya Inc. (State College, PA, USA). Vitamin E standards were obtained from Calbiochem (La Jolla, CA, USA). Fatty acid methyl ester standard mixture (FAME) Supelco 37 was obtained from Supelco (Bellefonte, PA, USA).

### 2.2. Sample Collection

Three samples of quince leaves with an origin in the village of Pereiro, municipality of Pinhel, Guarda, Portugal, were collected during the months of October, November, and December. This farm in Guarda (coordinates in WGS84: Lat: 40.725443 Long: −7.01789) is a biological plantation of 11,000 prolific cultivars of the variety *Cydonia vulgaris* Lusitanica that reached an actual peak of 46,000 Kg of quince fruits for the processing industry. It should be emphasized that this area is incorporated in a total production territory of 800 ha, with 400 ha of this being explored under biological conditions. No pesticides or herbicides are used to treat the plants, and the trees are not watered to achieve the best fruit possible according to the climate and soil conditions of the region. This particularity makes these cultivars a perfect source of samples for a reliable analysis and study of the healing properties of phytochemicals and nutrients identifiable in this plant, including quince leaves. This large-scale project of biological agriculture was the first candidate to be financed by the Financing Institute and Support of development of Agriculture and Fishing (IFADAP - *Instituto de Financiamento e Apoio ao Desenvolvimento da Agricultura e das Pescas*). Each sample was dried in a stove (Memmert UL6D, Germany) at 30 ± 2 °C for 5 days (in the dark). The mean drying yield was 49.82%.

### 2.3. Sample Preparation for ICP-MS Analysis

Quince leave samples were ground in a mill (GM200 GrindoMix, Retsch) before organic analysis. For inorganic evaluation, 0.2 g of dry leaf sample was digested using microwaves (MW) within a closed system at 170 °C using 1 mL of HNO_3_, 2 mL of H_2_O_2_, and 1 mL of H_2_O. After cooling, the vessel contents were transferred to volumetric flasks and the volume was made up with 25 mL of deionized water.

### 2.4. Moisture Content

Assessment of moisture content was determined in triplicate through infrared hygrometry readings at 105 °C using 1 ± 0.1 g of milled leaves sample (infrared balance, Scaltec model SMO01, Scaltec Instruments, Heiligenstadt, Germany).

### 2.5. Inorganic Elements Quantification

Mineral concentration was obtained by inductively coupled plasma mass spectroscopy (ICP-MS) on a Thermo ICP-MS X Series equipped with a Burgener nebulizer. The system functions with a plasma power at 1400 w, an argon flux of 13 ml/min, an auxiliary gas flux of 1 mL/min, and a sample flux of ~1mL/min. 

The tuning procedure was performed daily using a multielement solution (^6^Li, In, Ce, U, 10µg/L each) and the response for oxides (^140^Ce^16^O/^140^Ce ratio) did not exceed 2%.

External calibration was performed using multielement standard solution in 1% nitric acid (*v*/*v*) at the following element concentration levels: 0, 0.2, 0.4, 1.0, 2.0, 5.0, 10, 50, and 100 µg/L for minor constituents and 0, 0.02, 0.04, 0.1, 0.2, 0.5, 1,5, and 10 mg/l for major elements. The internal standard (10.0 µg/L ^115^In) was added on-line. This procedure was accomplished with three sample groups under different growing stages, i.e., green (October), yellow (November), and brown (December) leaves. Moreover, each sample group is the result of green, yellow, and brown leaves collected from six different trees within the respective month. For obtaining a biological average per group, a mix of several leaves was made, with an approximate weight from six trees following the chemical digestion in accordance with the method cited above. In the end, three different solutions were obtained, relating to the green, yellow, and brown sample groups. Each analysis was carried in triplicate readings per solution sample.

### 2.6. Extraction and Quantification of Phenolic Content (TPC)

For the extraction of phenolic phytochemicals, the mass/volume ratio was optimized using milled quince leaves and 80/20 methanol/water (*v*/*v*) as solvent. The mixture was agitated in a magnetic stirrer (MS-H-S10 magnetic stirrer, ChemLand, Poland) at a constant temperature (40 °C) and agitation (600 rpm) for 1 h, according to Melo et al. (2021) [41].

The total phenolic content (TPC) was determined by a spectrophotometric method with the Folin–Ciocalteu reagent at room temperature using an absorbance reading of 765 nm in a microplate reader (BioTek Instruments, Synergy HT GENS5, EUA) following Costa et al. (2018) [42]. Results are reported in g of gallic acid equivalents (GAE)/100 g of sample fresh weight.

### 2.7. Extraction of Lipid Fraction 

The lipid fraction extraction was performed as described by Melo et al. (2021) [41] using absolute ethanol and *n*-hexane (HPLC grade) as solvents in constant agitation (Multi Reax EU, Heidolph, Germany). The final extract (1 mL) was divided for vitamin E (500 μL) and fatty acid profile (the other 500 μL) assessment.

#### 2.7.1. Vitamin E Profile

For vitamin E profile determination, the final extract (using tocol as an internal standard) was injected in a HPLC-DAD-FLD (high performance liquid chromatography with diode array detector and fluorescence detector) system (Jasco, Tokyo, Japan) equipped with a MD-4015 multiwavelength diode array detector (Jasco, Tokyo, Japan), and an FP-4025 fluorescence detector (Jasco, Tokyo, Japan) programmed for an excitation of 290 nm, an emission of 330 nm, a PV-4180 pump, an AS-4050 autosampler, and a normal phase Supelcosil TM LC-SI column (75 mm × 3.0 mm, 3.0 μm, Supelco). The eluent was 1,2% 1,4-dioxane in *n*-hexane (*v*/*v*). The flow rate was 0.7 mL/min. The injection volume was 20 μL. Vitamin E isomers (α-tocopherol, β-tocopherol, γ-tocopherol, δ-tocopherol, α-tocotrienol, β-tocotrienol, γ-tocotrienol, and δ-tocotrienol) were identified based on their UV spectra and by comparison to the retention times of standards. Isomers were quantified based on the fluorescence signals. Results are expressed in mg/100 g of sample fresh weight.

#### 2.7.2. Fatty Acids (FA) Profile

For FA profile determination, a transmethylation with KOH in methanol was performed to the extract to obtain methyl esters, according to ISO 12966-2:2017 [43]. The obtained extract was then injected in a GC-FID system (Shimadzu, Tokyo, Japan) equipped with an AOC-20i automatic sampler and a split/splitless auto injector (Shimadzu, Tokyo, Japan) at 250 °C, a flame ionization detector (Shimadzu, Tokyo, Japan) at 270 °C, and a CP-Sil 88 silica capillary column (50.0 m × 0.25 mm inner diameter and 0.20 μm film thickness, Varian, Middelburg, Netherlands). The carrier gas was helium. The injection volume was 1 μL. The used program was: 120 °C held for 5 min, 2 °C/min to 160 °C held for 2 min, and 2 °C/min to 220 °C held for 10 min. Identification was performed by comparing the retention times of fatty acids methyl esters to a standard mixture (FAME 37, Supelco, Bellefonte, PA, USA). The data were analyzed based on relative peak areas. Results are expressed as relative percentage (%) of total FA.

### 2.8. Statistical Analysis

Statistical analysis was performed using IBM SPSS Statistics (v. 26 for Windows, IBM Corp., Armonk, 241 NY, USA). The evaluation of statistical significance was determined by ANOVA and Tukey’s HSD to assess significant differences between samples at a 5% significance level. 

## 3. Results

### 3.1. Inorganic Analysis

In this study, it was determined that the green leaf sample had a mineral content with a predominance of Ca 15000 mg, Mg 5500 mg, K 2200 mg, Si 880 mg, P 760 mg, Fe 119 mg, Na 100 mg; the yellow leaf sample had mineral content of Ca 17000 mg, Mg 4700 mg, K 2300 mg, Si 1800 mg, P 700 mg, Na 310 mg, Fe 109 mg; and the brown leaf sample had a mineral content of Ca 21480 mg, K 12010 mg, Mg 4350 mg, P 830 mg, Si 630 mg, Na 520 mg, Fe 40 mg by 1.0 Kg of substrate (Table 1). During maturation of Portuguese quince leaf, it is possible to notice an increase in the mineral content of Ca and K, and a slight decrease in Mg and Fe. On the other hand, after a significant increase in Si level following the transition from the green to yellow leaves, it is possible to verify a severe decrease in the content of Si in the brown leaves (Table 1). On the contrary, after a very small decrease in the levels of P during the transition period from green to yellow leaves, it is possible to verify a significant increase in the brown leaves (from 700mg to 830 mg/Kg) in comparison to the green leaves (Table 1).

### 3.2. Organic Analysis

The assessment of moisture content in the green quince sample showed a percentage of approximately 10% (Table 2), which is very similar to the yellow and brown quince samples and means that quince leaves did not suffer a significant change in their water content throughout their ripening period. With respect to the phenolic content the quince leaves, a total amount of gallic acid between 9–12 g per 100g of net weight of each sample was detected. A non-significant decrease in the relative weight of gallic acid equivalent (GAE)/100g was observed between the 11.51g of green leaf net weight and the 9.35g of yellow leaf net weight, indicating a loss in phenols throughout the ripening time, followed by a slight increase to 10.97g at full maturation in brown leaves (Table 2).

#### 3.2.1. Vitamin E Profile

**Figure 1:**
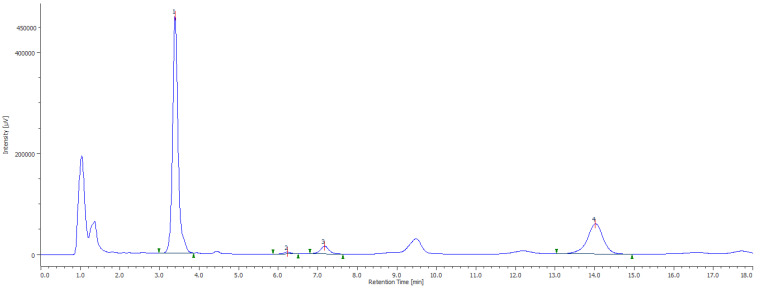
Example of a chromatogram obtained with fluorescence detection (FP-4025 fluorescence detector, Jasco, Tokyo, Japan) for the vitamin E profile of leaf samples of *Cydonia oblonga* Mill. assessed by HPLC-DAD-FLD (1—α-tocopherol, 2—β-tocopherol, 3—γ-tocopherol, 4—tocol/internal standard).

The study of the vitamin E profile allowed for the determination of the quince leaves’ composition of α, β, and **γ**-tocopherol. α-tocopherol, the only form known to meet human requirements, was the predominant compound in all green, yellow, and brown leaf samples. So, the total vitamin E composition decreased from a relative weight of 29 mg in green leaves to circa 13 mg/100 g net weight in yellow leaves. Based on this, it is possible to deduce that the vitamin E content decreases drastically with time from the ripening of green to yellow leaves, but is curiously followed by a significant rise back to 30.86 mg/100 g net weight in brown leaf (Figure 1, Table 2).

#### 3.2.2. Fatty Acid Profile

With respect to the fatty acid composition of quince leaves, it was observed that the general contents of SFA and MUFA showing a significant percentage increase from 44.58 ± 0.60 in green leaves to 56.32 ± 0.33 in yellow leaves and from 10.65 ± 1.13 in green leaves to 15.53 ± 0.85 in yellow leaves, with a close return to the first (green leaf) values in brown leaves, respectively (Figure 1, Table 2). On the contrary, the relative percentage of PUFA showed a significant percentage decrease from 44.77 ± 0.54 in green leaves to 28.15 ± 0.52 in yellow leaves, followed by a gain back to 40.74 ± 0.25 in the brown leaves (Figure 1, Table 2). 

**Figure 2:**
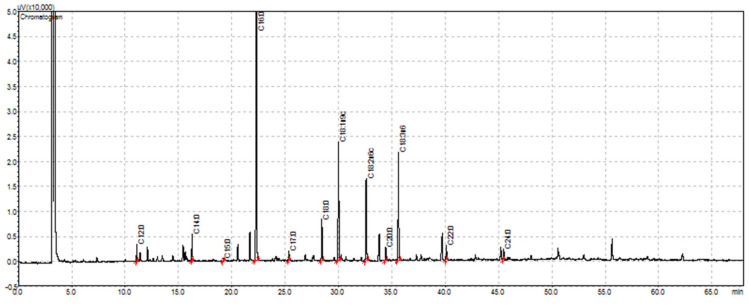
Example of a chromatogram obtained for the fatty acid profile of quince leaves assessed by GC-FID. C12:0—lauric acid, C14:0—myristic acid, C15:0—pentadecanoic acid, C16:0—palmitic acid, C17:0—margaric acid, C18:0—stearic acid, C18:1n9c—oleic acid, C18:2n6c—linoleic acid, C18:3n3—linolenic acid, C20:0—arachidic acid, C22:0—behenic acid, C24:0—lignoceric acid.

It was possible to identify the fatty acids that are largely present in this part of the plant—palmitic acid (C16:0, 30–38%) and linolenic acid (C18:3n3, 16–32%)—followed by the linoleic (C18:2n6c, 13%) and oleic acid (C18:1n9c, 11–16%) (Figure 1, Figure 2, Table 2). 

C18:3n-3 decreased from 32.20% in green leaves to a minimum of 15.57% in yellow leaves within a one-month period, and a gain up to 26.55% in brown leaves one month later (Table 2).

## 4. Discussion

### 4.1. Inorganic Analysis

Quince is an underrated fruit with proven significant nutritional qualities as it is a rich source of carbohydrates, fiber, proteins, vitamins, different organic acids, and minerals [44]. Quince fruit is reported to have a higher relative nutritional value in comparison to apples [15]. According to Bíró and Lindner 1999, the mineral content of quince (Na 9.2 mg, K 189 mg, Ca 66 mg, Mg 10 mg, Fe 1.1 mg, P 25 mg) is reported to be twice as that of apples (Na 2 mg, K 112 mg, Ca 5.5 mg, Mg 6 mg, Fe 0.3 mg, P 8 mg) [45]. The results (Table 1) show an increase in the mineral content of Ca and K and a slight decrease in Mg and Fe. It was also possible to observe a significant increase in Si content during the maturation of green leaves to yellow leaves, which was followed by a severe decrease in the Si content during the brown leaves period. 

Moreover, inorganic analysis showed a composition by Kg of leaves close to or above the DRI values for the following components in both green and yellow leaf samples: calcium, magnesium, iron, phosphorus, copper, and manganese. Comparing with the study of Bíró G and Lindner 1999, we can deduce that quince leaves are richer in inorganic content relative to both quince and apple fruits. According to the World Health Organization (WHO), malnutrition, defined as deficiencies, excesses, or imbalances in a person’s intake of energy and/or nutrients, acts as a double burden for consequences as wasting, stunting, low birth weight, and micronutrient deficiencies, on the one hand, and overweightness, obesity, and diet-related noncommunicable diseases (NCDs), on the other hand. Efforts have been made to overcome these serious world public health issues, namely the creation of sustainable processes for micronutrient-fortified foods such as rice and flours. Positive results by these measures have been already attained with the elimination of iodine deficiency disorders through universal salt iodization and the impressive results gained in the control and prevention of iron deficiency and derived anemia through fortification of flour with iron and folic acid [46]. The reason why the initiative was extended to the fortification of edible oils and fats with vitamin A and D was to overcome the widely prevalent forms of subclinical vitamin A deficiency disorders [47] as well of zinc, vitamins B2 and B12, niacin, and calcium deficiency. Chronic hypocalcemia may derive from several factors and affects several systems in the body, with osteoporosis being a main concern that can be prevented with a balanced diet and calcium supplements, sometimes in combination with vitamin D or magnesium supplementation as well, depending on the factor leading to low calcium levels [48]. The element magnesium is involved in more than 300 biochemical reactions within the body, and a healthy intake is crucial for the nerves’ and muscles’ functional statuses, heart beating, and bone formation. For example, a diet rich in proteins, calcium, and vitamin D requires higher levels of magnesium, the same way that hypomagnesemia might lead to metabolic disorders such as hypocalcemia and hypokalemia [47,49]. Malnutrition and/or malabsorption, obesity, and alcohol withdrawal can also lead to deficiencies in inorganic phosphate or adenosine triphosphate associated with disorders in the central and peripheral nervous system and myopathies that require the re-establishment of phosphorus levels [50]. Just as with zinc, copper is involved in cellular signaling pathways and has been mostly found in the human brain [51]. Both cupric and cuprous ions can cause oxidative stress and free radical damage, so the intracellular level of copper is controlled by binding to specific proteins such as Cu transporters and copper chaperones. Copper containing enzymes and transcription factors play a key role in the cell’s integrity, proliferation, and energy production, mainly in collaboration with iron absorption, mediating the production of red blood cells and keeping a healthy homeostatic function within bones, blood vessels, nerves, and the immune system [51,52]. Additionally, mechanisms related to copper have shown to be a prospective therapeutic target for conditions such as influenza A, lung inflammation, and cancer, in addition to neurodegenerative disorders such as Alzheimer’s and Parkinson’s disease as well as Menkes and Wilson’s diseases [53,54,55]. Moreover, during the last years, liver copper deficiency has been correlated with obesity and nonalcoholic fatty liver disease (NAFLD), for which prospective pharmacologically enhancing therapeutics have been explored as an approach to treat excessive weight and associated pathologies [56]. Another trace mineral with proven utility within human physiology and biochemistry is manganese. This mineral is involved in blood clotting and hemostasis in conjunction with vitamin K and also acts as a cofactor for several enzymes, including manganese superoxide dismutase, arginase, and pyruvate carboxylase, through which manganese is involved in amino acids, cholesterol, glucose, and carbohydrate metabolism; reactive oxygen species (ROS) scavenging; bone formation; reproduction; and immune response. The human body contains about 10 to 20 mg of manganese, of which 25% to 40% is in bone with the remaining being distributed between the liver, pancreas, kidney, and brain [57,58,59,60,61]. A very limited body of evidence in humans suggests that manganese deficiency might be related to: (1) impaired growth and bone formation in children, (2) skin rashes, (3) hair depigmentation, (4) diminished serum cholesterol, (5) changes in lipid and carbohydrate metabolisms, (6) changes in tolerance to glucose, (7) increased activity of alkaline phosphatase in men, and (8) mood changes or premenstrual pain in women [58,62]. According to our results, quince leaves acquire nutritional importance in the context of mineral deficiencies and might serve as cheap potential substrates for food enrichment and supplement manufacture, in particular, for addressing calcium, magnesium, iron, manganese, and phosphorus deficiencies. 

### 4.2. Organic Analysis

The moisture content (Table 2) of all green, yellow, and brown quince leaves was around 10%, without significant differences (*p* > 0.05), revealing that the samples did not suffer a significant change in their water composition during ripening. Unlike moisture, this period might have influenced other parameters such as, for instance, acidity, which is probably more dependent on phenolic compounds and FA composition than on water. Gallic acid, a secondary polyphenolic metabolite, is a well-known natural antioxidant used in the pharmaceutical industry as a standard for determining the TPC of various analytes, using the Folin–Ciocalteu assay. Tea leaves are considered to be an important source of gallic acid, containing up to 4.5 g/kg fresh weight [63]. The TPC results (Table 2) varied between 9–12 g GAE/100 g of quince leaves. The sample of yellow leaves presented the lowest content (9 g GAE/100 g), indicating a significant (*p* > 0.05) loss in these compounds throughout the ripening period. In the previous comparative study of Costa et al. (2009), quince leaves exhibited a significantly higher reducing power in relation to green tea leaves. The Costa group found that under the oxidative action of 2,2’-azobis (2-amidinopropane) dihydrochloride (AAPH), quince leaf methanolic extract significantly protected the erythrocyte membrane from hemolysis similarly to green tea (IC50 = 30.7 and 24.3 μg/mL, respectively, *p* > 0.05). These findings highlight the potential of quince leaves for the prevention of cardiovascular disorders and mitigation of risk based on cardioprotective and hypolipidemic properties as previously described [31,39,64]. Khademi et al. (2013) found a significant reduction in serum lipids as well a similar thickness of atheroma in both the control and intervention groups, with animals treated with a methanolic fraction of leaf extract [37], confirming the potential of the traditional use of the plant to treat metabolic and cardiovascular diseases [65,66]. Several studies have already shown the efficacy of quince fruits and leaf extracts in the treatment and prevention of atherosclerosis, endothelial dysfunction, hypertension, diabetes, and hyper-homocysteinemia [16,64,67,68,69,70,71,72,73,74,75]. Once again, despite the slight loss in phenolic content during its ripening from green to brown, all of Pinhel’s Portuguese quince leaves emerge as a phytochemical alternative to the use of statins, which are prioritized for the treatment of hypercholesteremia and currently known for its risks of muscular toxicity. Studies conducted in the past few years by Silva and coworkers [31,35,39,76,77,78,79] have demonstrated that *Cydonia oblonga* Mill. is a good, safe, and low-cost natural source of different classes of phenolic compounds, such as flavonol and flavone heterosides and, especially, caffeoylquinic acids. Polysaccharides of quince fruit inhibited the activity of tyrosine phosphatase (IC50 = 2.07 μg/mL), indicating its capability to treat type 2 diabetes and obesity [80]. Moreover, quince is used in sore throat, cough, pneumonia, and lung disease [2,7,37,81,82,83,84,85]. The antimicrobial potential of quince leaf extract was also tested by Benzarti et al. (2018), who tested the activity against eight pathogenic bacteria using the disc diffusion method. The quince leaf extract inhibited *Enterococcus faecium* (ATCC 19434), *Streptococcus agalactiae*, and *Bacillus subtilis* [86], opening a window for important future research exploring the antimicrobial properties of the extract against infections other than airway-related infections. Carvalho et al. (2010) used quince leaf and fruit methanolic extracts to assess their ability to inhibit the growth of human renal (A-498 and 769-P) and colon (Caco-2) cancer cell lines, showing a concentration-dependent growth inhibitory activity toward human colon cancer cells. Quince leaves contain the highest content of polyphenols of quince parts, mainly caffeoylquinic acids and quercetin and kaempferol derivatives [1], possibly responsible for the remarkable antiproliferative activity of quince leaf extract against colon cancer cells. It is possible to infer that rich polyphenol quince leaf extract may have pharmaceutical potential to intercept or to mitigate oxidative stress-induced damage leading to cancer [86]. The vitamin E profile (Table 2) revealed that quince leaves present α-, β-, and γ-tocopherols. The major isomer identified in all of the leaves’ samples—with a higher relative percentage of green and brown leaves compared to yellow leaves—was α, which is the only isoform known to meet human requirements. α-tocopherol can be incorporated into biological membranes, not only preventing protein oxidation and inhibiting lipid peroxidation, but also maintaining cell membrane integrity—especially those with high amounts of polyunsaturated fatty acids (PUFA)—while also protecting against cell damage as well as inhibiting the activity of protein kinase C (PKC) and PKC-mediated pathways. Moreover, it is able to inhibit the activity of powerful inflammatory mediators induced by PKC such as COX-2 and IL-8 in many tissues [87], resulting in a decrease in prostaglandin production [88,89]. Vitamin E is also known to suppress the transcription of the vascular endothelial growth factor (VEGF or VEGF-A) gene responsible for expressing an important angiogenic protein, thereby restraining angiogenesis and tumor development [90]. A study with male smokers suggested that VEGF-A levels decreased during an intervention where randomized men received a trial α-tocopherol supplement compared to those who received a placebo [91]. Yellow leaves present a significant decrease in total vitamin E, almost a third of total amount determined in green and brown leaves. Thus, green and brown quince leaves seem more valuable as a potential substratum for pharmaceutical and cosmetic industries, where tocopherols may be used to help recover hair and skin damage and promote healthy aging. A biomaterial named D-α-tocopheryl polyethylene glycol succinate (TPGS), used in the development of various drug delivery systems (e.g., micelles, liposomes, and other nanoparticles), can function as a solubilizer, emulsifier, additive, and permeability enhancer as well as an absorption enhancer. Additionally, TPGS is capable of overcoming multidrug resistance mediated by the P-gp efflux pump [92]. Because of this, quince leaf extracts can be an asset in further drug development in pharmaceutical industry, improving colloidal stability and in vivo antitumor activity. Furthermore, α-tocopherol is known to be a safe food additive [93], and so the relative content of this metabolite in quince leaves’ extract turns it into an affordable option for use in the food industry. Studies indicate that vitamin E supplementation may be a valuable addition to the treatment of patients with seasonal allergic rhinitis [94], and the substantial vitamin E content in quince leaves supports that this substrate is a potential low-cost solution for the production of natural vitamin E supplements. Moreover, it helps justify the efficient and wide traditional use of quince leaf extract to treat allergic rhinitis and asthma [82,95,96]. Lastly, with the increasing incorporation PUFAs such as linoleic acid and α-linolenic acid (ALA) in contemporary diets, a higher intake of vitamin E is required, as α-tocopherol is a very important lipophilic antioxidant able to break peroxidation chains [97]. Even though no final value for the human recommended daily intake of α-tocopherol has been yet defined, an estimated value of the requirements has been based on the equation 0.4–0.6 mg of RRR-α-tocopherol/g of PUFA [98]. PUFA peroxidation chains compromise the integrity of cellular membrane components essential for embryological development and lifelong neuromuscular acuity, which reinforces the relevance of vitamin E protection, so a higher intake in diets by incorporation of more vegetable oils, nuts, or seeds or by supplementation with nutraceuticals is advisable. Thus, quince leaves might be an accessible source for the development of dietary supplements. From a comparison between the FA content in the yellow and green leaf samples, it is possible to see that ALA significantly (*p* > 0.05) decreased by half within one month of ripening from green to yellow leaves, rising again in the following month (brown leaves). As the FA results are presented in relative percentage due to the decrease in ALA, the other FA percentages increase, which happened for palmitic, oleic, and myristic acids. According to the Academy of Nutrition and Dietetics, the energy provided by dietary fats in healthy adults should account for 20–25% of the total energetic intake, with a major preference for unsaturated over saturated fatty acids (SFA), as the differences in their chemical composition result in diverse physiological pathways and metabolic functions [99]. The impact of ALA on health has been studied by several groups in animals and humans and a review on its benefits and properties points to the following: (1) inhibition of MCF-7 breast tumor growth in mice; (2) apoptosis of hepatoma cells in rats; (3) decrease in depression-like behavior and stimulation of functional stroke recovery in mice; (4) improvement of the brain’s blood circulation and vasodilation of the basilar artery and protective effects against stroke in mice and rats; (5) production of optimal levels of DHA in the brains of postweaning rats; (6) anti-inflammatory effects in both acute and chronic arthritis in rats; (7) significant improvement in bronchial asthma in patients; (8) improvement of atopic dermatitis in atopic patients; (9) decreased levels of total cholesterol, low-density lipoprotein (LDL), LDL/HDL ratio, and serum triglycerides in mild hypertensive patients; (10) decline of hot flash activity in women; (11) effectiveness in primary and secondary prevention of coronary artery disease in risky patients; (12) antiarrhythmic therapeutic actions in in smoking women; (13) decrease in serum cholesterol in hypercholesteric patients; (14) decreased systolic blood pressure, total cholesterol, LDL-C, and insulin levels in patients with metabolic syndrome; (15) partial modulation of inflammation and endothelial activation in mildly hyperlipidemic patients; and (16) decrease in the risk of mild dementia in elderly patients [100]. According to the same authors, oral consumption of ALA (α- linolenic acid) has been verified to be safe so far, though attention has been paid to the observational, yet inconclusive studies where ALA could be possibly linked with prostate carcinogenesis and macular degeneration. Major dietary sources of ALA are whole flaxseed and flaxseed oil, which have been associated with gastrointestinal complaints such as bloating, flatus, and stomach aching. Having said that, the significant ALA content found in both the green and brown quince leaf extract makes them an alternative source of this essential plant-based n-3 FA, which is the substrate for the synthesis of more unsaturated n-3 fatty acids formed by longer chain eicosapentaenoic acid (C20:5n3) and docosahexaenoic acid (DHA, C22:6n3), essential for tissue function in both men and women, though the conversion to DHA is higher in the female gender, possibly due to the action of estrogen [101,102]. As previously mentioned, ALA, as a PUFA, can produce lipid peroxidation products under exposure to air or UV radiation, which may produce adverse effects if not appropriately controlled, and which can be prevented through radicals scanvenging with vitamin E [103]. It would be interesting to study a strategy for the supplement of ALA enriched with protective α-tocopherol, both derived from quince leaf extract, given the fact that these substrates appear to be a substantial source of both. Considering that, similar to the ALA content, the vitamin E value was higher for green and brown quince leaves, it would be useful to merge the biological activity of both green and brown quince leaves, creating a functional synergy that promotes the antioxidant and cytoprotective activities of α-tocopherol with enhancement of the anti-inflammatory effect of ALA. In this study, after palmitic acid and ALA, the most predominant FAs are linoleic and oleic acids. Oleic acid (n-9) is the most common monounsaturated fatty acid (MUFA) in diets. It is partially essential because the body is not able to fully compensate its absence by synthetizing it in diets low in this FA [104]. Both palmitic and oleic acid are non-esterified fatty acids (NEFAs), where palmitic acid is a saturated chain and oleic acid is a monounsaturated chain. NEFAs are associated with three key concepts of metabolic syndrome: obesity, insulin resistance (IR), and type 2 diabetes mellitus (T2DM). However, palmitic acid and oleic acid seem to contribute differently for IR. Vessby et al. (2001) reported that insulin sensitivity improves with changes in the quality of the FA intake, with a reduction in SFA and increase in MUFA within a fat intake up to 37% of the total energy [105]. Additionally, a higher percentage of total MUFA, in contrast with the total intake of SFA, is considered as a shielding factor against pancreatic cancer [106]. Distinct mechanisms are implicated in the action of oleic acid over insulin sensitivity, such as countering the reduction in the AMP-activated protein kinase (AMPK) activity induced by palmitic acid. AMPK is the action target of the most prescribed drug for T2DM: metformin [107]. Studies demonstrated a similar action between the drug metformin and oleic acid by hampering the detrimental effects of AMPK activation with palmitic acid, which ultimately leads to the prevention of endoplasmic reticulum stress, inflammation processes, and IR [108,109]. This evidence implicates that oleic acid is a potential alternative to metformin in the treatment of IR implicated in metabolic syndromes and T2DM after discontinuation of treatment due to adverse effects of metformin such as nausea, diarrhea, loss of appetite, and xerostomia, among others [110]. Regarding the studies in this area and the favorable results of oleic acid content in the brown leaf sample, we propose that quince leaves are an inexpensive source of this biological material. On the other hand, FAs also hold a crucial position in brain function and the peripheral nervous system as an energy source, molecule signaling agents, and as pillars of the integrity of the cellular membrane. Evidence has related FAs with neurodegenerative diseases, mental disorders such as depression, stroke, and trauma. Once again, the dietary quality of FAs is correlated with the exposition to disease: while SFA and n-6 PUFA are considered to be injurious to nervous system function, MUFA and n-3 PUFA are acknowledged to be beneficial. Moreover, an optimized health status would be reached with a 1:1 intake proportion of n-6 PUFA/n-3 PUFA. This ratio has been disrupted in the consumption habits of modern society, therefore triggering negative effects such as neuroinflammation and loss of memory associated with Alzheimer’s and Parkinson diseases [111]. Even though the WHO advises a higher ratio of 5:1 to 10:1 for n-6 to n-3 PUFA, such as that found in olive oil, contrarily to other vegetable oils except for flaxseed and soy oils [112], in our results, it is possible to observe a ratio ranging from 0.41 (green leaves) to 0.58 (brown leaves). On the other hand, previous studies analyzed the addition of olive leaves (3%) to Tunisian olive cultivars before the oil extraction, which increased peroxide value, phenol and tocopherol contents, and oxidative stability. The conclusions were positive, with the resultant olive oil being an extra virgin olive oil with appreciated sensory properties [113]. Taking into account that Tunisian olive oil is higher in linoleic and palmitic acids than oleic acid [112], adding olive leaves showed a rather economic and efficient solution to meet both goals: harmonized FA composition and sharpened sensory properties. So, following the same line of thought, the n-6/n-3 ratio of quince leaves shows them to have the potential to be used as a fortifier of n-3 PUFAs in vegetable oils if further studies verify that their organoleptic properties are suitable for proper acidity and taste. Overall, green and brown leaf samples presented a different profile, characterized by a higher value (55.42% and 55.26%, respectively) of unsaturated fatty acids when compared with yellow leaf composition (43.68%), originating a ratio of 1.3 and 1.2, respectively, which is in line with the recommended nutritional guidelines of a PUFA/SFA ratio > 0.4 [111,112]. Given the tendency of ALA for a pro-oxidant state, it is possible to infer that yellow quince leaves, due to their close MUFA content (15.53%) to PUFA (28.15%) content, have a higher potential to be used as an extract for meat preservation to defend against dietary dangers such as cancer development and immune system compromise [114,115,116,117]. Of course, in order to obviate and support the potential therapeutic indications with *C. oblonga*, it will be necessary to carry out deep specific clinical studies as well as bio-physiological evaluations based on adequate cell culture in the future. So, one simple, expeditious, and functional hypothesis, supported by intelligence-based medicine, consists of a screening method using electro-biological data from natural compounds using a computerized bioresonance integrator, MORA Nova [118,119,120,121]. This integrator mechanism allows us to check and describe the physiological role of natural drugs in different human systems. Thus, it is possible to create a virtual and personalized human model from the collection of functional physiological parameters from various human systems diagnosis and store it in memory in the intelligent integrator. This human virtual model is submitted to a specific fluid or liquid interface to evaluate the respective interference from dynamic substances (molecules, antibodies, drugs, fluids, plants, etc.) as a therapy effect or, on the contrary, as a toxic influence. Our preliminary in silico assays using this methodology showed that immunological alterations correlate with the severity of manifestations of diseases such as contagious infections, cancer, autism, hypertension, and depression, among many others. To improve those conditions, an immune-boosting effect might be achieved by combined biological and artificial intelligence-based treatments. With effect, it was also verified to have a different reaction intensity depending on the leaf maturation stage. Green leaves seem to regulate and harmonize hyperfunction, whereas yellow and brown leaves enhance the orthopathy in hypodynamic dysfunctions. On the other hand, a mixed effect occurs due to the combination of green and yellow/brown leaves, giving a more balanced and corrected therapy concerning several physiological parameters when tested in less-severe dysfunction (i.e., when unhealthy people’s deviations from healthy values are not greater than 10 points up or down). In fact, electric conductance measurements have evidenced *C. oblonga*’s relevant role in the regulation of systemic immunity that seems to be potentiated by combined treatments of individualized electromagnetic frequencies with phytotherapeutic activated decoctions or infusions. A deeper and statistical evaluation with different people under specific physiological conditions and/or diseases when submitted to treatments of *C. oblonga* collected in different maturation periods is suggested. Then, a similar program is possible and pertinent to apply to the cell *in vitro* culture at different pathological conditions using green, yellow, brown, or mixed leaves, combined or not with electromagnetic frequencies from Mora Nova. Concerning this bioresonance methodology, several deeper studies are already underway to better evaluate the properties of *C. oblonga* in the therapeutic clinic setting.

## 5. Conclusions

Natural compounds from plants have an important role in pharmaceutical research and may result in the discovery of effective pathway modulations. Bioactives endowed with nutraceutical properties might act either by positive preconditioning or confer therapeutic value against preexisting diseases. Regarding the mineral content of Pinhel’s Portuguese quince leaf extract, it would be interesting to further explore the potential of this substrate for possible therapeutic supplementation or for food fortification with calcium, potassium, magnesium, iron, copper, manganese, and phosphorus. Furthermore, organic analysis showed interesting results. In this study, not only the presence of phenolic was confirmed, but as well as that of organic acids and vitamin E in biological green, yellow, and brown Pinhel’s Portuguese quince leaves. In other natural sources such as honey and grape seeds, cardioprotective effects have been attributed to various available agents such as polyphenols and flavonoids [122,123,124]. For this, the quince leaf may be a potential source of protective agents against diseases associated with an inflammatory process. Cardiovascular diseases are associated with diabetes, high blood pressure, atherosclerosis, heart inflammation, and blood clotting, all of which are physiological states derived from oxidative stress produced by ROS [125,126,127,128]. Moreover, because of their richness in polyphenols, with values superior to the content identified in tea leaves, derivatives from quince leaves may be a safe and low-cost alternative to (1) hypercholesteremia treatment by statins, (2) pharmaceutical approaches to cease or to reduce oxidative stress-induced damage leading to cancer, and (3) antimicrobial therapeutics against Gram-positive bacterial infections of airways, among others. Being a considerable source of vitamin E, namely of the potent antioxidant and anti-inflammatory α-tocopherol, quince leaf extract is a potential substratum for pharmaceutical, cosmetic, and food industries where it can be proposed (1) as a possible alternative to the production of vitamin E supplements, (2) as a source of tocopherol esters to recover hair and skin damage and settle a healthy aging, (3) for further drug development in pharmaceutical industry improving colloidal stability and greater in vivo antitumor activity, and (4) as a safe food additive. Regarding FA composition, the major compounds identified in this study were palmitic acid and linoleic acids, and given the potential nutraceutical properties of ALA and its considerable content in the green and brown leaf extracts, this substrate can be an alternative to flaxseed seeds and oil in ALA supplements with the standing feature of an enriched protective effect of vitamin E over PUFA stabilization in membranes. Regarding the nutraceutical properties of oleic acid, quince leaves can also be tested as a potential source for an alternative use to metformin against the deleterious effects of IR implicated in contemporary disorders that carry a burden on worldwide health system costs, such as T2DM and metabolic syndromes. Nevertheless, the optimal n-6/n-3 FA ratio found in quince leaves also shows the potential to be used as a complement of n-3 PUFA in vegetable oils that lack the 1:1 ratio found in olive oil known to protect from inflammatory damage in nervous and cardiovascular systems. Particularly, yellow quince leaves represent a potential source of FA for the preservation of food such as pork and beef meat, owing to their balanced PUFA and MUFA contents. Overall, quince leaves demonstrated a wide range of potential for applications in the food, pharmaceutical, and cosmetic industries. Finally, due to the current drastic and prolonged COVID-19 pandemic situation, and based on global physiological concerns, mainly cardiovascular, pulmonary, and immunological defenses, it has already been verified by our preliminary in silico studies with green, yellow, and brown quince leaves that it is possible to preview an excellent supplement for a complementary therapy. So, further research is needed for a deeper understanding of the nutraceutical power of this economical vegetable source with great potential for industrial purposes.

## Figures and Tables

**Table 1:** Mineral analysis of leaf samples of *Cydonia oblonga* Mill—green leaves in October, yellow leaves in November, and brown leaves in December. Each sample group was made by a mix of different tree leaves to obtain an approximate biological average. As a result, present results express a mean ± reading deviation (*n* = 3) which varies between a 3.0–5.0% gap. ** DRIs (daily recommended intake)—https://www.nap.edu (accessed on 30 October 2021).

	Samples	Units	Green Leaves (October)	Yellow Leaves (November)	Brown Leaves (December)	DRIs—Male (31–50 Y.O.) **
Elements						
**As**		mg/Kg	<3.00	<3.00	<3.00	N/A
**Ba**		mg/Kg	50.00 ± 0.05	49.00 ± 0.05	51.00 ± 0.05	N/A
**Ca**		g/Kg	15.00 ± 0.05	17.00 ± 0.05	21.48 ± 0.03	1.00 g/d
**Cd**		mg/Kg	<0.25	<0.25	<0.25	N/A
**Cr**		mg/Kg	<0.50	<0.50	<0.50	0.04 mg/d
**Cu**		mg/Kg	3.90 ± 0.03	3.50 ± 0.03	0.69 ± 0.03	0.90 mg/d
**Fe**		mg/Kg	119.00 ± 0.05	109.00 ± 0.05	40.00 ± 0.05	8.00 mg/d
**K**		g/Kg	2.20 ± 0.03	2.30 ± 0.03	12.01 ± 0.05	3.40 g/d
**Mg**		g/Kg	5.50 ± 0.03	4.70 ± 0.03	4.35 ± 0.03	0.42 g/d
**Mn**		mg/Kg	52.00	50.00	85.00	2.30 mg/d
**Na**		g/Kg	0.10 ± 0.03	0.31 ± 0.03	0.52± 0.03	1.50 g/d
**Pb**		mg/Kg	<2.50	<2.50	<2.50	N/A
**Se**		mg/Kg	<2.50	<2.50	<2.50	0.06 mg/d
**Sr**		mg/Kg	78.00 ± 0.05	71.00 ± 0.05	79.00 ± 0.05	N/A
**Zn**		mg/Kg	27.00 ± 0.05	21.00 ± 0.05	10.00 ± 0.03	14.00 mg/day
**Mo**		mg/Kg	<3.00	<3.00	<3.00	0.05 mg/d
**Si**		g/Kg	0.88 ± 0.03	1.8 ± 0.03	0.63 ± 0.03	N/A
**P**		g/Kg	0.76 ± 0.03	0.70 ± 0.03	0.83 ± 0.03	0.70 g/d

**Table 2:** Organic analysis of leaf samples of *Cydonia oblonga*. Results expressed as mean ± standard deviation (*n* = 3). Different letters denote significant differences (*p* > 0.05). TPC—total phenolic content, GAE—gallic acid equivalents, C12:0—lauric acid, C14:0—myristic acid, C15:0—pentadecanoic acid, C16:0—palmitic acid, C17:0—margaric acid, C18:0—stearic acid, C18:1n9c—oleic acid, C18:2n6c—linoleic acid, C18:3n3—linolenic acid, C20:0—arachidonic acid, C22:0—behenic acid, C24:0—lignoceric acid, SFA—saturated fatty acids, MUFA—monounsaturated fatty acids, PUFA—polyunsaturated fatty acids.

	Samples	Green Leaves (October)	Yellow Leaves (November)	Brown Leaves (December)
Elements				
**Moisture** (%)		10.60 ± 0.46 ^a^	10.35 ± 0.29 ^a^	10.16 ± 0.10 ^a^
**TPC** (g GAE/100 g)		11.51 ± 0.54 ^a^	9.35 ± 0.19 ^b^	10.97 ± 0.57 ^a^
**α-Tocopherol** (mg/100 g)		29.16 ± 0.89 ^a^	12.50 ± 1.20 ^b^	29.87 ± 1.00 ^a^
**β-Tocopherol** (mg/100 g)		0.24 ± 0.00 ^b^	0.20 ± 0.01 ^c^	0.26 ± 0.00 ^a^
**γ-Tocopherol** (mg/100 g)		0.44 ± 0.01 ^b^	0.65 ± 0.05 ^a^	0.73 ± 0.01 ^a^
**Total vitamin E** (mg/100 g)		29.84 ± 0.90 ^a^	13.34 ± 1.26 ^b^	30.86 ± 0.99 ^a^
**C12:0** (%)		1.10 ± 0.03 ^c^	1.96 ± 0.06 ^a^	1.34 ± 0.07 ^b^
**C14:0** (%)		2.03 ± 0.10 ^b^	3.49 ± 0.08 ^a^	2.09 ± 0.01 ^b^
**C15:0** (%)		0.21 ± 0.03 ^b^	0.33 ± 0.01 ^a^	0.14 ± 0.01 ^c^
**C16:0** (%)		31.25 ± 0.48 ^b^	38.05 ± 0.14 ^a^	30.27 ± 0.31 ^b^
**C17:0** (%)		0.87 ± 0.01 ^b^	1.25 ± 0.03 ^a^	0.80 ± 0.02 ^c^
**C18:0** (%)		4.10 ± 0.07 ^c^	5.69 ± 0.05 ^a^	4.59 ± 0.08 ^b^
**C18:1n9c** (%)		10.65 ± 1.13 ^b^	15.53 ± 0.85 ^a^	14.52 ± 0.76 ^a^
**C18:2n6c** (%)		12.57 ± 0.03 ^b^	12.58 ± 0.26 ^b^	14.20 ± 0.29 ^a^
**C20:0** (%)		1.66 ± 0.10 ^b^	1.98 ± 0.08 ^a^	1.74 ± 0.11 ^a,b^
**C18:3n3** (%)		32.20 ± 0.55 ^a^	15.57 ± 0.33 ^c^	26.55 ± 0.29 ^b^
**C22:0** (%)		1.92 ± 0.07 ^a^	2.02 ± 0.08 ^a^	2.10 ± 0.13 ^a^
**C24:0** (%)		1.44 ± 0.01 ^b^	1.55 ± 0.05 ^a,b^	1.65 ± 0.07 ^a^
**SFA** (%)		44.58 ± 0.60 ^b^	56.32 ± 0.33 ^a^	44.74 ± 0.63 ^b^
**MUFA** (%)		10.65 ± 1.13 ^b^	15.53 ± 0.85 ^a^	14.52 ± 0.76 ^a^
**PUFA** (%)		44.77 ± 0.54 ^a^	28.15 ± 0.52 ^c^	40.74 ± 0.25 ^b^
